# A Novel Boron Dipyrromethene-Erlotinib Conjugate for Precise Photodynamic Therapy against Liver Cancer

**DOI:** 10.3390/ijms25126421

**Published:** 2024-06-11

**Authors:** Wenqiang Wu, Chengmiao Luo, Chunhui Zhu, Zhengyan Cai, Jianyong Liu

**Affiliations:** 1 China State Institute of Pharmaceutical Industry, Pudong New Area, Shanghai 201203, China; skysnine@163.com; 2 Key Laboratory of Molecule Synthesis and Function Discovery, Fujian Province University, College of Chemistry, Fuzhou University, Fuzhou 350108, China; l403186011@163.com (C.L.); yuxun12181@163.com (C.Z.); 3 State Key Laboratory of Photocatalysis on Energy and Environment & National & Local Joint Biomedical Engineering Research Center on Photodynamic Technologies, College of Chemistry, Fuzhou University, Fuzhou 350108, China

**Keywords:** photosensitizer, photodynamic therapy, BODIPY, reactive oxygen species, erlotinib

## Abstract

Photodynamic Therapy (PDT) is recognized for its exceptional effectiveness as a promising cancer treatment method. However, it is noted that overexposure to the dosage and sunlight in traditional PDT can result in damage to healthy tissues, due to the low tumor selectivity of currently available photosensitizers (PSs). To address this challenge, we introduce herein a new strategy where the small molecule-targeted agent, erlotinib, is integrated into a boron dipyrromethene (BODIPY)-based PS to form conjugate **6** to enhance the precision of PDT. This conjugate demonstrates optical absorption, fluorescence emission, and singlet oxygen generation efficiency comparable to the reference compound **7**, which lacks erlotinib. In vitro studies reveal that, after internalization, conjugate **6** predominantly accumulates in the lysosomes of HepG2 cells, exhibiting significant photocytotoxicity with an IC_50_ value of 3.01 µM. A distinct preference for HepG2 cells over HELF cells is observed with conjugate **6** but not with compound **7**. In vivo experiments further confirm that conjugate **6** has a specific affinity for tumor tissues, and the combination treatment of conjugate **6** with laser illumination can effectively eradicate H22 tumors in mice with outstanding biosafety. This study presents a novel and potential PS for achieving precise PDT against cancer.

## 1. Introduction

Photodynamic therapy (PDT) is an innovative treatment option for an array of cancers and non-cancerous conditions [1,2,3]. It operates through the synergistic action of a photosensitizer (PS), light of a specific wavelength that matches the PS’s absorption spectrum, and molecular oxygen (^3^O_2_). Upon exposure to light, the PS is excited to an excited singlet state (S1) from its ground state and then undergoes intersystem crossing (ISC) to arrive at an excited triplet state (T1). The PS in this state can transfer energy to ^3^O_2_ present in the surrounding tissues, resulting in the production of highly reactive singlet oxygen (^1^O_2_), a process known as the type II mechanism. Alternatively, the PS in T1 can also directly transfer an electron to ^3^O_2_ or other biological molecules, generating free radical species that subsequently react with other cellular components, leading to the formation of O_2_^·−^, H_2_O_2_, and other free radicals, a process that is regarded as type I mechanism. These reactive oxygen species (ROS) can cause oxidative damage to cellular components, resulting in cell death via necrosis or apoptosis. In practice, the type II mechanism is more dominant than the type I mechanism, with most currently available PSs, such as porphyrins and phthalocyanines, operating on this principle [4]. It is noteworthy that the short lifespan (ranging from 30 to 180 nanoseconds) and limited migration distance (less than 100 nanometers) of the ROS ensure that PDT is only effective within the irradiated area by light, which endows PDT with high spatial selectivity [5]. This precision is a significant advantage of PDT, as it allows for targeted treatment of tumors while minimizing damage to adjacent healthy tissue. Moreover, PDT also features minimal invasiveness, insignificant multidrug resistance, and a broad spectrum of antitumor activity, gaining widespread attention in recent years [6,7,8]. However, the poor tumor specificity of most clinically used PSs can result in skin photosensitivity and damage to healthy tissues. As a result, patients often need to avoid sunlight exposure and remain in a dark room for an extended period after PDT [9], which can be mentally taxing. To mitigate these issues, several strategies have been implemented to enhance the specific accumulation of PS, thereby improving PDT efficacy while reducing systemic photosensitivity. One such strategy involves conjugating a PS with functional biomolecules like aptamers [10,11], peptides [12,13,14], antibodies [15,16], or small molecule targeting moieties such as biotin [17], saccharides [18,19,20], and folic acid [21], which can significantly increase the tumor specificity of PS. Another approach is the use of nanomaterials as carriers for PS delivery to tumor tissues [22,23,24,25]. Additionally, activatable PSs represent an alternative strategy to enhance PDT selectivity [26,27]. These PSs are initially inactive but become active upon encountering endogenous stimuli specific to tumors, such as elevated levels of glutathione (GSH) [28,29,30] and hydrogen peroxide [31,32], overexpressing enzymes [33,34,35,36,37], as well as a weak acidic environment [38,39,40].

Recently, the conjugation of PSs with small-molecule target-based anticancer drugs has attracted much attention [41,42]. These conjugates combine the high phototoxicity of the PSs with the outstanding specificity of the small-molecule targeted agent. Epidermal growth factor receptor (EGFR) overexpression is a hallmark observed across a spectrum of tumors and is associated with tumor cell proliferation, angiogenesis, invasion, metastasis, and inhibition of apoptosis [43]. This overexpression makes EGFR a significant target for cancer therapy. Erlotinib, a small-molecule EGFR tyrosine kinase inhibitor, works by binding to the ATP-binding site of the EGFR dimer, thereby inhibiting the receptor’s kinase activity. It has demonstrated specificity for tumor cells that overexpress EGFR, which is a common characteristic in many types of cancer. Boron dipyrromethene (BODIPY) dyes are characterized by their strong absorptions in the visible spectrum, exceptional photostability, and ready chemical modifications [44,45]. These attributes render them highly versatile for a spectrum of applications [46,47,48] including PSs in the field of photodynamic therapy. In this study, we choose erlotinib as the specific target for conjugation with a BODIPY-based PS through a triethylene glycol chain, which endows the conjugate with enhanced amphiphilicity, thus benefitting cellular uptake. As expected, this novel BODIPY-Erlotinib conjugate has shown a marked selectivity to HepG2 liver cancer cells and H22 tumors in the mice, correlating with its substantial anticancer potency in both in vitro and in vivo settings. To the best of our knowledge, there are few reports regarding the conjugation of small molecule targeted anticancer agents with BODIPY-based PSs for targeted PDT.

## 2. Results and Discussion

### 2.1. Synthesis

**Scheme 1:**
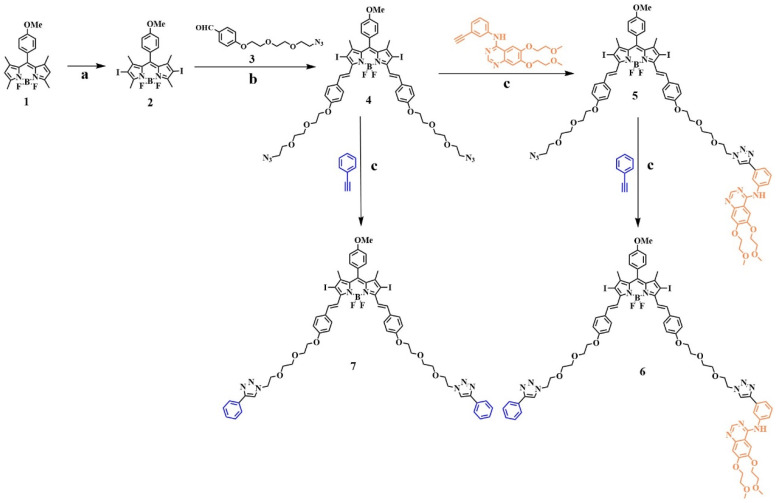
Synthesis of BODIPY-Erlotinib conjugate **6** and the reference compound **7**. Reagents and conditions: (a) I_2_, HIO_3_, ethanol, 60 °C; (b) Acetic acid, piperidine, toluene, reflux, 2 h; (c) Phenylacetylene, CuSO_4_·5H_2_O, sodium ascorbate, chloroform-ethanol-water.

Scheme 1 shows the synthetic route used to prepare BODIPY-Erlotinib conjugate **6** as well as the reference compound **7** without an erlotinib unit. Firstly, two iodine atoms were grafted to the BODIPY core to enhance intersystem crossing, which resulted in higher ^1^O_2_ generation efficiency for PSs instead of emitting fluorescence. Two styryl groups were then conjugated into the BODIPY ring by a Knoevenagel reaction to expand the π-conjugated system, giving rise to a redshift absorption falling in the optical therapeutic window that minimizes the light absorption and scattering by normal tissues, thereby improving therapeutic efficiency. The triethylene glycol chains provided the hydrophobic rigid BODIPY ring with a hydrophilic structure. Finally, Copper(I)-Catalyzed Azide-Alkyne Cycloaddition (CuAAC) was applied to combine diazide-substituted BODIPY **4** with erlotinib, resulting in the formation of the BODIPY-Erlotinib conjugate **5**. The remaining azido group in conjugate **5** further underwent CuAAC with phenylacetylene, yielding BODIPY-Erlotinib conjugate **6**. Diazide-substituted BODIPY **3** was treated with an excess of phenylacetylene to produce compound **7**, which served as a reference to demonstrate the targeting ability of the erlotinib conjugate. The characterization of all the new compounds was performed using ^1^H NMR and ^13^C NMR spectroscopy, along with high-resolution mass spectrometry (Figures S5–S15).

### 2.2. Spectroscopic and Photosensitizing Properties

**Figure 1:**
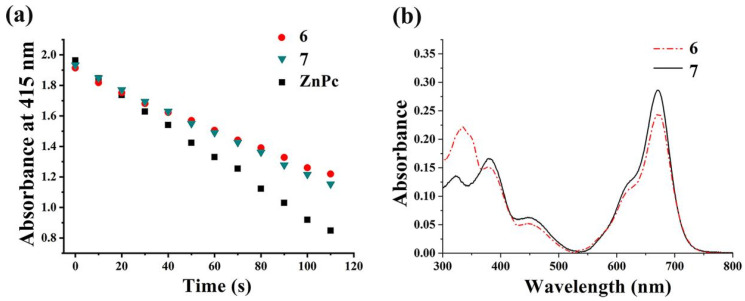
(**a**) Degradation of DPBF in the presence of **6** and **7** under light irradiation. (**b**) Electronic absorption spectra of **6** and **7** in the DMEM culture medium (both at 5 μM).

The electronic absorption and fluorescence emission spectra of conjugate **6** and the reference compound **7** were examined in dimethylformamide (DMF), and the corresponding data were compiled in Table 1. The results revealed that both compounds exhibited a strong Q-band absorption at 662 nm, and the absorption peaks strictly adhered to the Beer-Lambert law under experimental concentrations (Figure S1), and their logε reached approximately 5.00, indicating that the BODIPY derivatives possessed a high molar extinction coefficient, a characteristic of ideal PSs. The two PSs displayed a similar fluorescence emission peaking at 693 nm under excitation at 610 nm, and their fluorescence quantum yields were measured to be 0.24 and 0.22, respectively, using unsubstituted zinc(II) phthalocyanine (ZnPc) as the reference (Table 1). Additionally, 1,3-diphenylisobenzofuran (DPBF) was employed as ^1^O_2_ indicator to evaluate the photosensitizing ability of **6** and **7**. As demonstrated in Figure 1a, conjugate **6** combined with 660 nm laser irradiation could efficiently induce oxidative degradation of DPBF as a result of significantly decreased absorbance at 415 nm, and its singlet oxygen quantum yield (Φ_∆_) was detected to be 0.32 with reference to ZnPc (Table 1), which is comparable to that of **7**. The findings implied that the introduction of erlotinib did not affect the photophysical- and photochemical properties of the BODIPY-based PS.

BODIPYs featuring extensive conjugated systems are prone to aggregation when present in biological media, a phenomenon that can result in a broadening of the Q-band absorption, a notable reduction in fluorescence emission, and diminished photosensitizing efficacy. To evaluate the aggregative tendencies of compounds **6** and **7** in a biological context, their electronic absorption and fluorescence emission spectra were measured within Dulbecco’s Modified Eagle’s Medium (DMEM). The observation of sharp and intense Q-band absorption (Figure 1b) coupled with robust fluorescence emission (Figure S2) suggested that both PSs remained largely non-aggregated in DMEM. This minimal aggregation is a beneficial trait for their potential use in fluorescence imaging and photodynamic therapy.

Next, we conducted an assessment of the fat-solubility by determining the octanol-water partition coefficients of compounds **6** and **7**. As expected, the incorporation of erlotinib enhances the lipophilic nature of BODIPY, as evidenced by the higher logP value exhibited by compound **6** in comparison to compound **7** (6.66 versus 6.01). Furthermore, the pharmacokinetic properties of **6** and **7** have also been predicted using the SwissADME webserver [49]. The data are summarized in Table S1. The same conclusion, “compound **6** has a higher logP value than compound **7**”, is obtained from the theoretical calculations. A variance of approximately 15% between the experimental and calculated logP values exists, falling within an acceptable and reasonable range.

### 2.3. In Vitro Study

**Figure 2:**
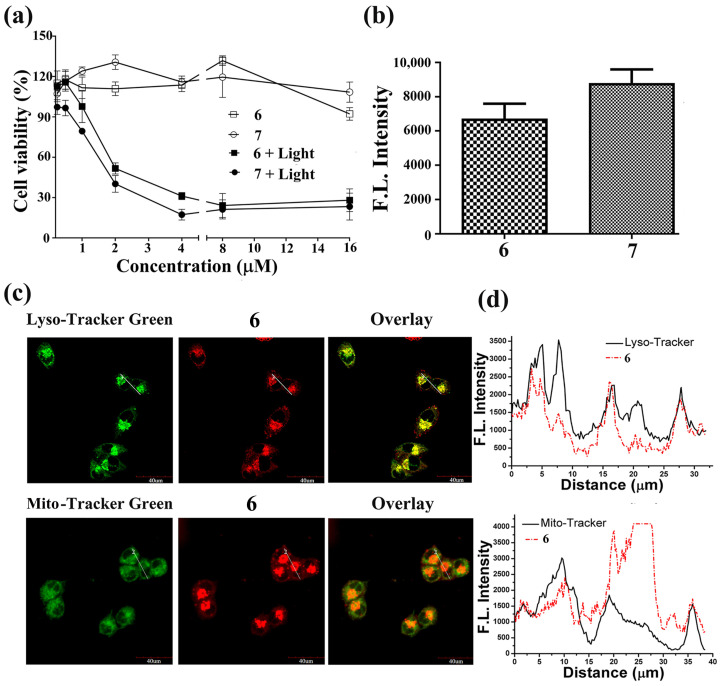
In vitro photobiological activity investigations: (**a**) Cytotoxicities of **6** and **7** against HepG2 cells with or without light irradiation; (**b**) ROS generation efficiency of **6** and **7** in HepG2 cells by using DCFH-DA as the probe; (**c**) Confocal laser scanning microscopy (CLSM) images of HepG2 cells for Lyso-Tracker and BODIPY-based PS **6**; (**d**) Fluorescence intensity profiles of **6** (red line) and Tracker (black line) traced along the white lines in the merged images in (**c**).

Encouraged by the prominent optical properties of conjugate **6**, we explored its photobiological behaviors in human liver cancer HepG2 cells overexpressing EGFR in comparison with those of the control **7**. Initially, the cytotoxicity of conjugate **6** together with **7** against HepG2 cells was examined by MTT assay. It could be seen that the viability of HepG2 cells is nearly 100% with only light illumination (Figure S3), indicating the light dosage used in this study is safe. As reflected in Figure 2a, both BODIPY dyes exhibited insignificant cytotoxicity even up to 16 µM in the absence of light. After the treatment of PSs combined with light irradiation (1.5 J/cm^2^), the cell viability substantially decreased in a concentration-dependent manner. The IC_50_ value of conjugate **6** was calculated to be 3.01 μM, which is comparable to that of the reference compound **7** (IC_50_ = 1.95 μM). The relatively high photodynamic activities of these two PSs were attributed to their high ^1^O_2_ generation efficiency and low aggregations in the DMEM culture medium.

The activation of PSs leads to the production of ROS, which are considered the primary agents responsible for cell death in PDT. Typically, an increased level of ROS correlates with enhanced photocytotoxicity. In this context, the efficiency of ROS generation by both BODIPYs against HepG2 cells was examined by using 2′,7′-dichlorodihydrofluorescein diacetate (DCFH-DA) as a ROS indicator. DCFH-DA is non-fluorescent and can freely cross the cell membrane to be hydrolyzed into 2′,7′-dichlorodihydrofluorescein (DCFH), which is further oxidized by intracellular ROS into strong fluorescent 2′,7′-dichlorofluorescein (DCF). Therefore, quantifying the fluorescence of DCF at 525 nm can indirectly reflect the ability of a PS to generate ROS. As presented in Figure 2b, The ROS generation efficiency of conjugate **6** is slightly lower than that of **7**, which aligns with their ^1^O_2_ quantum yields in DMF and their observed in vitro photocytotoxicity.

The PDT-generated ^1^O_2_ has an extremely short half-life and diffusion radius in biological systems. Hence, the subcellular localization of PSs uptaken by cancer cells is crucial for PDT treatment efficacy. HepG2 was first treated with BODIPY-Erlotinib conjugate **6** for 24 h and then stained with commercially available probes LysoTracker DND-26 for 60 min or MitoTracker Green FM for 30 min, respectively. As depicted in Figure 2c, the fluorescence of LysoTracker (excited at 488 nm, monitored at 510–570 nm) well overlapped with the fluorescence emitted by **6** (excited at 633 nm, monitored at 650–750 nm). Conversely, the fluorescence of MitoTracker (excited at 488 nm, monitored at 510–570 nm) was not superimposed with that of **6**. The same phenomenon was also observed from the trace lines (Figure 2d). The results suggested that conjugate **6** is mainly distributed in the lysosomes after internalization into the HepG2 cells via endocytosis.

**Figure 3:**
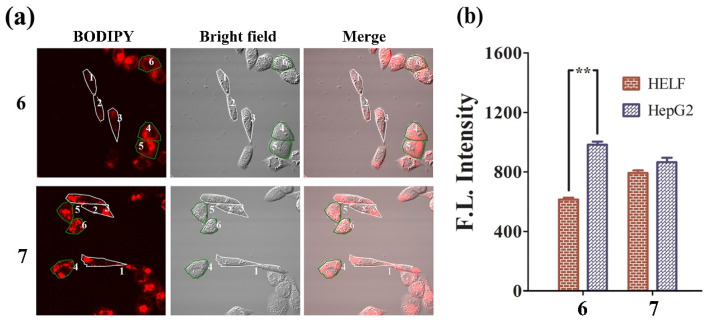
Competitive uptake investigations: (**a**) CLSM images of HepG2 and HELF cells treated with **6** or **7** (both at 5 μM) for 24 h in the same culture dish (1, 2, and 3 are the HELF cells; 4, 5, and 6 are the HepG2 cells); (**b**) Comparison of relative intracellular average fluorescence intensity of BODIPY in HepG2 and HELF cells. Data are expressed as means ± SD. Statistical significance ** (*p* < 0.01).

A competitive cellular uptake experiment was conducted to evaluate the specific affinity of conjugate **6** to cancerous cells. Briefly, we co-cultured HELF cells, human embryonic lung fibroblasts that express low levels of EGFR, and HepG2 cells, which are morphologically distinct from HELF cells, in a single cell culture dish. Following a 24-h incubation with BODIPYs, the fluorescence emitted by compound **6** or **7** (both excited at 633 nm, with emission monitored between 650 and 750 nm) was documented using confocal laser scanning microscopy (Figure 3a). The fluorescence intensity from **6** in the HepG2 cancer cells was significantly higher compared to that in HELF cells. In contrast, there was no significant difference in fluorescence between HELF and HepG2 cells for the reference compound **7** (Figure 3b). These findings suggested that BODIPY **6** bearing an erlotinib moiety is capable of specifically targeting cancer cells that overexpress EGFR.

### 2.4. In Vivo Studies

**Figure 4:**
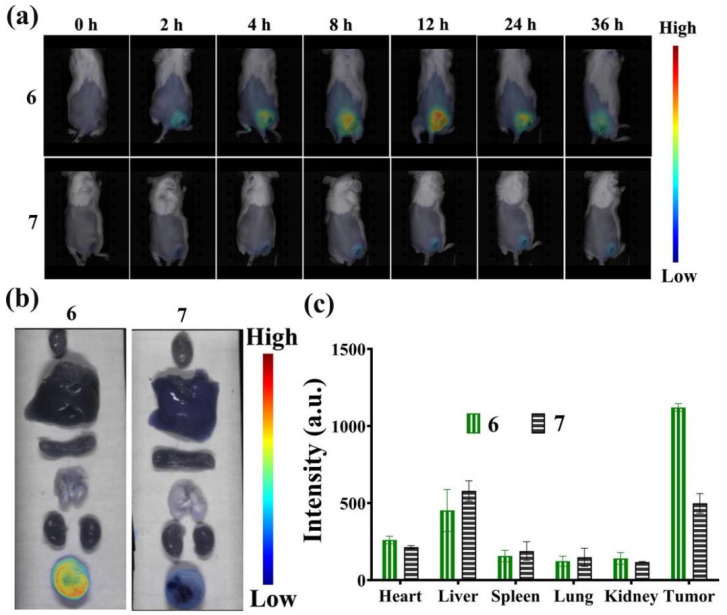
(**a**) In vivo fluorescence images of PSs in the H22 tumor-bearing mice after administration at various post-injected times; (**b**) Ex vivo fluorescence images of PSs in tumors and major organs excised from the mice; (**c**) quantitative results of (**b**).

To further investigate the tumor-targeting capacities of conjugate **6**, in vivo fluorescence imaging was conducted by using the fluorescence molecular tomography (FMT) technique. Firstly, PSs (**6** and **7**) were administered to the mice with H22 tumors via the tail vein, and then their fluorescence images on tumor tissues with time were recorded to reveal the drug accumulation process. The results indicated that conjugate **6** exhibited a time-dependent increase in tumor accumulation, with a peak observed at 12 h post-administration (Figure 4a). Notably, the reference compound **7** demonstrated significantly weaker fluorescence intensity in the tumor tissues compared to conjugate **6** over the whole period (Figure 4a). To further ascertain the distribution of the PSs within the body, mice were euthanized at 12 h post-injection, and major organs and tumors were collected for ex vivo fluorescence imaging. The data revealed that when mice were treated with conjugate **6** (Figure 4b), there was a pronounced fluorescence signal in the tumor, with minimal fluorescence observed in other organs. In contrast, when treated with the erlotinib-free compound **7**, fluorescence signals were obtained mainly in the tumor and liver. Additionally, a more pronounced intratumoral fluorescence was observed in mice treated with conjugate **6** compared to those treated with compound **7** without an erlotinib unit. These findings suggested that the inclusion of the erlotinib component in BODIPY significantly enhanced the specificity of the compound toward tumors, resulting in greater accumulation within the tumor sites. This enhanced specificity is attributed to the erlotinib molecule, which appears to play a pivotal role in the preferential targeting of tumor tissues.

**Figure 5:**
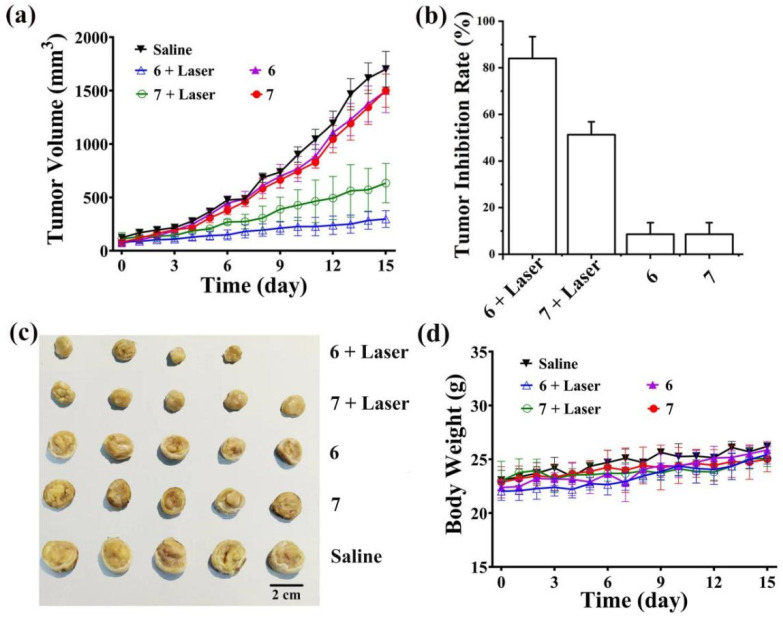
In vivo antitumor efficacy evaluations: (**a**) Tumor growth profiles; (**b**) Tumor growth inhibition rate; (**c**) Photographs of the excised tumors after 15 days of treatment; (**d**) Changes in body weight during treatment.

Next, the therapeutic efficacy of the BODIPY-Erlotinib conjugate **6**, and the reference compound **7** was evaluated using H22 tumor-bearing mice over a 15-day inspection. The mice were randomly assigned to one of five treatment groups (*n* = 5 per group): (i) Saline control; (ii) 6 alone, (iii) 6 plus laser irradiation; (iv) 7 alone; (v) 7 plus laser irradiation. The tumor volumes of each group were continuously measured for 15 days. As illustrated in Figure 5a, the tumors in mice treated solely with either **6** or **7** exhibited a rapid growth rate akin to the saline control group, indicating that the two BODIPY dyes alone had no significant tumor-suppressing effect without laser stimulation. However, when combined with laser irradiation, both PSs demonstrated a significant inhibition of tumor growth. Notably, the BODIPY-Erlotinib conjugate **6**, achieved a higher tumor growth inhibition compared to compound **7** (Figure 5b), which lacks the erlotinib unit. This outcome was further corroborated by the visual representation of tumor photographs in Figure 5c. Furthermore, no substantial changes in body weight were observed throughout the treatment period (Figure 5d), indicating the good biosafety profile of conjugate **6**. Additionally, the treatment involving conjugate **6** and laser irradiation did not cause any discernible damage to the heart, liver, spleen, lung, or kidney (Figure S4), suggesting that PS **6** does not induce systemic toxicity. Collectively, these findings indicated that conjugate **6** offered a potent antitumor effect with minimal side effects, positioning it as a candidate of great promise for precise PDT.

## 3. Materials and Methods

### 3.1. General

Details regarding the purification of solvents, reagents, apparatus, cell culture, and animal feeding can be found in the Electronic Supplementary Materials. Compounds **1** and **2** were synthesized following the established procedures that have been previously documented [50].

### 3.2. Synthesis

#### 3.2.1. Synthesis of Diazide-Substituted BODIPY **4**

The mixture of BODIPY **2** (0.31 g, 0.5 mmol), azide-substituted aldehyde **3** (0.29 g, 1.0 mmol), acetic acid (2.0 mL, 34.9 mmol), piperidine (2.4 mL, 24.3 mmol), and a trace of magnesium perchlorate in toluene (90 mL) was stirred under reflux for 24 h with the assistance of a Dean-Stark apparatus to efficiently remove any water generated during the reaction. The reaction mixture was then concentrated under reduced pressure. The residue was subsequently poured into silica gel and eluted by a mixture of ethyl acetate and dichloromethane (1:15, *v*/*v*). The diazide-substituted BODIPY **4** (0.35 g) was obtained as a green solid in 60% yield. ^1^H NMR (400 MHz, CDCl_3_): δ (ppm) = 8.13 (d, *J* = 16.8 Hz, 2 H), 7.60 (d, *J* = 8.8 Hz, 4 H), 7.59 (d, *J* = 16.8 Hz, 2 H), 7.17 (d, *J* = 8.8 Hz, 2 H), 7.04 (d, *J* = 8.8 Hz, 2 H), 6.97 (d, *J* = 8.4 Hz, 4 H), 4.20 (t, *J* = 4.8 Hz, 4 H), 3.88–3.92 (m, 7 H), 3.75–3.79 (m, 4 H), 3.67–3.73 (m, 8 H), 3.40 (t, *J* = 4.8 Hz, 4 H), 1.50 (s, 6 H); ^13^C NMR (101 MHz, CDCl_3_): δ (ppm) = 160.55, 159.96, 150.33, 145.77, 138.96, 138.71, 133.30, 129.77, 129.65, 129.23, 127.21, 116.85, 115.04, 114.86, 82.70, 70.93, 70.75, 70.11, 69.79, 67.59, 55.43, 50.73, 17.74; HRMS (ESI): *m*/*z* calculated for C_46_H_49_BF_2_I_2_N_8_NaO_7_ [M+Na]^+^: 1151.1775, found 1151.1773.

#### 3.2.2. Synthesis of BODIPY-Erlotinib Conjugate **5**

Diazide-substituted BODIPY **3** (0.33 g, 0.30 mmol) and erlotinib (0.12 g, 0.30 mmol) were initially dissolved in a mixed solvent of chloroform-ethanol-water (12:1:1, *v*/*v*/*v*, 14 mL). Subsequently, crushed CuSO_4_·5H_2_O (45 mg, 0.18 mmol) and sodium ascorbate (72 mg, 0.36 mmol) powder were introduced into the solution. The mixture was continuously stirred at ambient temperature for 24 h, then extracted with chloroform (3 × 90 mL) and water (90 mL). The organic layers collected were condensed. The residue was subjected to silica gel column chromatography using a mixture of dichloromethane and methanol (15/1, *v*/*v*) as the eluent to afford a green solid **5** (0.19 g) in 42% yield. ^1^H NMR (400 MHz, CDCl_3_): δ (ppm) = 8.62 (s, 1 H), 8.30 (s, 1 H,), 8.09 (d, *J* = 16.8 Hz, 1 H), 8.05 (s, 1 H), 7.94 (d, *J* = 16.8 Hz, 1 H), 7.89 (d, *J* = 8.4 Hz, 1 H), 7.64 (br s, 1 H), 7.59 (d, *J* = 16.8 Hz, 1 H), 7.53 (d, *J* = 8.0 Hz, 2 H), 7.50 (d, *J* = 8.8 Hz, 1 H), 7.48 (s, 1 H), 7.34–7.41 (m, 3 H,), 7.22 (d, *J* = 8.4 Hz, 2 H), 7.16 (d, *J* = 8.0 Hz, 2 H), 7.06 (d, *J* = 8.4 Hz, 2 H), 6.86 (d, *J* = 8.4 Hz, 2 H), 6.70 (d, *J* = 8.8 Hz, 2 H), 4.60 (t, *J* = 4.8 Hz, 2 H), 4.25 (t, *J* = 4.8 Hz, 2 H), 4.14 (t, *J* = 4.8 Hz, 2 H), 4.08 (t, *J* = 4.4 Hz, 2 H), 4.05 (t, *J* = 4.8 Hz, 2 H), 3.94 (t, *J* = 4.8 Hz, 2 H), 3.91 (s, 3 H), 3.83–3.88 (m, 4 H), 3.80 (t, *J* = 4.4 Hz, 2 H), 3.65–3.76 (m, 12 H), 3.47 (s, 3 H), 3.42 (s, 3 H), 3.38 (t, *J* = 4.8 Hz, 2 H), 1.50 (s, 3 H), 1.44 (s, 3 H); ^13^C NMR (101 MHz, CDCl_3_): δ (ppm) = 160.62, 159.99, 159.79, 156.34, 154.18, 153.37, 150.35, 150.27, 148.71, 147.56, 146.96, 146.95, 145.91, 145.80, 139.79, 139.15, 138.71, 133.24, 131.36, 129.78, 129.62, 129.56, 129.40, 129.20, 129.16, 127.16, 121.36, 120.99, 120.91, 118.54, 116.67, 116.66, 115.05, 114.87, 109.46, 108.46, 102.35, 82.67, 82.63, 70.91, 70.80, 70.74, 70.60, 70.55, 70.09, 69.79, 69.62, 69.41, 68.84, 68.31, 67.61, 67.53, 59.27, 59.24, 55.46, 50.74, 50.39, 29.73, 17.74, 17.70; HRMS (ESI): *m*/*z* calculated for C_68_H_73_BF_2_I_2_N_11_O_11_ [M+H]^+^: 1522.3647, found 1522.3650.

#### 3.2.3. Synthesis of BODIPY-Erlotinib Conjugate **6**

Similarly, Treatment of azide **5** (0.15 g, 0.10 mmol) with phenylacetylene (41 mg, 0.40 mmol), CuSO_4_·5H_2_O (15 mg, 0.06 mmol), and sodium ascorbate (24 mg, 0.12 mmol) to produce conjugate **6** (0.15 g) as a green solid in 89% yield. ^1^H NMR (400MHz, CDCl_3_): δ (ppm) = 8.61 (s, 1 H), 8.31 (s, 1 H), 8.07 (d, *J* = 16.8 Hz, 1 H), 8.04 (s, 1 H), 7.97 (s, 1 H), 7.95 (d, *J* = 16.8 Hz, 1 H), 7.87 (d, *J* = 8.8 Hz, 1 H), 7.79 (vd, *J* = 7.6 Hz, 2 H), 7.73 (br s, 1 H), 7.59 (d, *J* = 16.8 Hz, 1 H), 7.56 (d, *J* = 16.8 Hz, 1 H), 7.45–7.51 (m, 3 H), 7.38 (d, *J* = 7.2 Hz, 2 H), 7.36 (d, *J* = 7.2 Hz, 2 H), 7.30 (d, *J* = 7.2 Hz, 1 H), 7.22 (d, *J* = 8.4 Hz, 2 H), 7.19 (br s, 1 H), 7.06 (d, *J* = 8.8 Hz, 2 H), 6.78 (d, *J* = 8.8 Hz, 2 H), 6.68 (d, *J* = 8.8 Hz, 2 H), 4.58 (t, *J* = 4.8 Hz, 2 H), 4.56 (t, *J* = 4.8 Hz, 2 H), 4.26 (t, *J* = 4.8 Hz, 2 H), 4.07 (t, *J* = 4.4 Hz, 4 H), 4.03 (t, *J* = 4.8 Hz, 2 H), 3.87–3.94 (m, 7 H), 3.85 (t, *J* = 4.4 Hz, 2 H), 3.76–3.82 (m, 4 H), 3.62–3.73 (m, 10 H), 3.48 (s, 3 H), 3.41 (s, 3 H), 1.51 (s, 3 H), 1.46 (s, 3 H); HRMS (ESI): *m*/*z* calculated for C_76_H_79_BF_2_I_2_N_11_O_11_ [M+H]^+^: 1624.4104, found 1624.4084.

#### 3.2.4. Synthesis of the Reference Compound **7**

Similarly, diazide-substituted BODIPY **5** (0.23 g, 0.22 mmol) was treated with phenylacetylene (0.10 g, 1.0 mmol), CuSO_4_·5H_2_O (15 mg, 0.06 mmol), and sodium ascorbate (24 mg, 0.12 mmol) to afford **7** (0.23 g) as a green solid with a yield of 87%. ^1^H NMR (400 MHz, CDCl_3_): δ (ppm) = 8.12 (d, J = 16.4 Hz, 2 H), 7.97 (s, 2 H), 7.82 (d, J = 7.2 Hz, 4 H), 7.59 (d, J = 16.4 Hz, 2 H), 7.56 (d, J = 8.8 Hz, 4 H), 7.30–7.41 (m, 6 H), 7.18 (d, J =8.4 Hz, 2 H), 7.05 (d, J = 8.8 Hz, 2 H), 6.87 (d, J = 8.8 Hz, 4 H), 4.58 (t, J = 4.8 Hz, 4 H), 4.10 (t, J = 4.8 Hz, 4 H), 3.91 (t, J = 4.8 Hz, 4 H), 3.90 (s, 3 H), 3.81 (t, J = 4.8 Hz, 4 H), 3.64–3.71 (m, 8 H), 1.51 (s, 6 H); ^13^C NMR (101 MHz, CDCl_3_): δ (ppm) = 160.69, 159.93, 150.50, 147.76, 145.91, 139.05, 138.85, 133.41, 130.91, 129.91, 129.76, 129.30, 128.96, 128.19, 127.35, 125.78, 121.05, 117.03, 115.06, 114.97, 82.75, 70.74, 69.79, 69.62, 67.62, 55.53, 50.47, 17.80; HRMS (ESI): *m*/*z* calculated for C_62_H_61_BF_2_I_2_N_8_NaO_7_ [M+Na]^+^: 1355.2716, found 1355.2706.

### 3.3. In Vitro Investigations

#### 3.3.1. Cytotoxicity Assessments

HepG2 cells were seeded onto 96-well plates at 6000 cells per well and incubated for 24 h. PSs (**6** or **7**) were diluted to the needed concentrations and added to six replicate wells. After incubation in a 5% CO_2_ incubator at 37 °C for 24 h, the medium was discarded, and fresh medium (100 μL) was added. Each well was then irradiated with a 660 nm LED lamp (1.5 J/cm^2^) and incubated for an additional 24 h. Subsequently, 10 μL of MTT working solution was added to each well and incubated for 4 h. The supernatant was then removed, and 100 μL of DMSO was added, followed by thorough mixing. The absorbance at 570 nm was measured using a microplate reader. Parallel experiments were conducted with drug addition but without light exposure to serve as the dark cytotoxicity. The measured data were subjected to nonlinear fitting to obtain the dose-dependent curves of cell viability using GraphPad Prism 5.0 analysis software, with the data expressed as Mean ± SD. The half-maximal inhibitory concentration (IC_50_) values of PSs against HepG2 cells were determined from the curves, reflecting the inhibitory capacity of different PSs on tumor cells. The experiments were repeated in triplicate.

#### 3.3.2. Intracellular ROS Measurements

The action mechanism of the ROS detection kit involves a probe, 2′,7′-dichlorodihydrofluorescein diacetate (DCFH-DA), which is non-fluorescent and can freely cross the cell membrane to be hydrolyzed into 2′,7′-dichlorodihydrofluorescein (DCFH). DCFH, in turn, cannot cross the cell membrane. When DCFH encounters intracellular ROS, it is oxidized into the fluorescent compound 2′,7′-dichlorofluorescein (DCF). Quantifying the fluorescence of DCF can indirectly reflect the ability of the PS to generate ROS. Briefly, the cells were seeded on a 96-well transparent plate at a density of 30,000 cells per well and cultured 24 h. Then, fresh medium containing PS (5 μM, containing 0.04% CEL and 1% DMSO) was added, and the cells were incubated for another 24 h in the dark. After washing three times with PBS to remove unabsorbed PS, 50 μL of the ROS probe was added to each well and incubated for 20 min. After washing three times with PBS, serum-free and phenol red-free medium was added, and the cells were irradiated by an LED lamp with a light dose of 1.5 J/cm^2^ and then incubated for 10 min. Finally, 100 μL of 1% SDS lysis buffer was added and shaken for 10 min, and the fluorescence intensity of DCF at 525 nm for each well was measured using a multifunctional microplate reader (excited at 488 nm). The experiments were performed in triplicate, and the data are expressed as the Mean ± SD.

#### 3.3.3. Subcellular Localization

Approximately 8000 HepG2 cells were plated on a confocal dish (diameter = 35 mm). After incubation of 24 h, the DMEM medium was removed, and the cells were rinsed three times with PBS, then filled with fresh medium containing PS (5 μM, containing 0.04% CEL and 1% DMSO) and incubated for another 24 h. The cells were washed with PBS three times and treated with MitoTracker@ Green FM (Invitrogen, Waltham, MA, USA, 2 μM in culture medium) for 30 min or LysoTracker@ Green DND-26 (invitrogen, 0.25 μM in culture medium) for 60 min. The culture medium was replaced with fresh serum-free and phenol red-free DMEM culture medium. Subsequently, the intracellular fluorescence images were captured by using an Olympus FV1000 Confocal Laser Scanning Microscope (Olympus Instrument Co., Ltd., Shinjuku-ku, Tokyo, Japan). The subcellular localization of the dyes was revealed by comparing the intracellular fluorescence images caused by the fluorescent probe and BODIPY-based PS.

#### 3.3.4. Competitive Uptake Examination

Cell suspensions of HepG2 and HELF were prepared at a concentration of 8 × 10^4^ cells/mL. Equal volumes (0.5 mL) of both cell suspensions were combined in a single culture dish. After allowing the cells to incubate overnight at 37 °C in a 5% CO_2_ incubator, the culture medium was discarded. Subsequently, the culture medium bearing PSs (5 μM, containing 0.04% CEL and 1% DMSO) was added, and the cells were incubated for an additional 24 h. Following this, the cells were washed three times with PBS to remove any residual PS. Then, serum-free and phenol red-free culture medium was added to the cells. The intracellular fluorescence of BODIPY was subsequently visualized using a Confocal Laser Scanning Microscope. By comparing the BODIPY fluorescence intensity within HepG2 and HELF cells, the competitive uptake of **6** and **7** by the two cell types was elucidated.

### 3.4. In Vivo Studies

#### 3.4.1. In Vivo Fluorescence Imaging

The tumor-bearing mouse models were established by subcutaneously implanting 1 × 10^6^ mouse-derived liver cancer H22 cells in the dorsal region of female ICR mice aged 5–6 weeks. The mice were randomly divided into three groups. Each consisted of three mice, with tumors allowed to approximately 100 mm^3^. Solutions of **6** and **7** were prepared in saline (100 μM, containing 0.04% CEL and 1% DMSO), and 100 μL solutions were intravenously administered to the experimental groups, respectively. Saline was administered to the control group. Fluorescence imaging was acquired by an in vivo fluorescence molecular tomography (FMT) imaging system (FMT2500LX, Perkin Elmer, Waltham, MA, USA) at various time points post-administration. Twelve hours after the intravenous injection via the tail vein, the mice were humanely euthanized. Subsequently, the heart, liver, spleen, lungs, kidneys, and tumor tissues were extracted. The excised organs and tissues were photographed using the FMT imaging system to document the distribution of the fluorescence.

#### 3.4.2. In Vivo Antitumor Activities Evaluation

For the assessment of in vivo tumor growth suppression, an additional 25 healthy mice were evenly allocated into 5 groups following the same protocol. The mice received intravenous injections of **6**, **7**, saline, **6**, and **7**. Each injection consisted of a 200 μL sample at a concentration of 100 μM. For the first two groups of mice, the tumor region was exposed to a 660 nm laser (200 mW/cm^2^) for 10 min, 12 h post-injection. The body weight and tumor volume of the mice were monitored daily. On day 15, the mice were euthanized, and the tumors along with the major organs were excised. These tissues were then subjected to histological examination using hematoxylin and eosin staining.

## 4. Conclusions

In conclusion, we have successfully synthesized a BODIPY-Erlotinib dyad, designated as conjugate **6**, by integrating the small molecule-targeted agent erlotinib into a BODIPY dye via a triethylene glycol linker. This novel compound is specifically engineered to enhance the precision of PDT for cancer treatment. In vitro optical investigations have demonstrated that the incorporation of erlotinib does not significantly alter the photophysical and photochemical characteristics of the conjugate, including its electronic absorption, fluorescence emission, and singlet oxygen production capacity. Competitive uptake experiments have shown that conjugate **6** with an erlotinib unit can specifically localize in HepG2 cancer cells that overexpress EGFR over HELF normal cells, which is not present for compound **7** lacking an erlotinib component. After being internalized into HepG2 cancer cells, this conjugate mainly localizes in the lysosomes and displays substantial photocytotoxicity with an IC_50_ value as low as 3.01 µM under light irradiation of 1.5 J/cm^2^. The outstanding tumor-targeting ability of this conjugate has also been verified in H22-tumor-bearing mice. The combined application of conjugate **6** with laser illumination is found to effectively eliminate H22 tumors while maintaining good biosafety. The observations suggest that conjugate **6** holds considerable potential as a targeted antitumor agent for precise photodynamic therapy.

## Figures and Tables

**Table 1:** Photophysical- and photochemical data of PSs **6** and **7** in DMF.

Compound	$\lambda_{max}^{abs}$/nm (logε)	$\lambda_{max}^{em}$/nm ^a^	Φ_F_ ^b^	Φ_∆_ ^b^
**6**	662/4.94	693	0.22	0.32
**7**	662/4.97	693	0.24	0.35

^a^ Excited at 610 nm; ^b^ Using ZnPc in DMF as the reference (Φ_F_ = 0.28, Φ_∆_ = 0.56).

## Data Availability

The authors declare that all the data obtained in this study can be provided on request.

## Supplementary Materials

The following supporting information can be downloaded at https://www.mdpi.com/article/10.3390/ijms25126421/s1.
