# Supplementary material for: A Novel Boron Dipyrromethene-Erlotinib Conjugate for Precise Photodynamic Therapy against Liver Cancer
Source: Int J Mol Sci. 2024 Jun 11;25(12):6421. doi: 10.3390/ijms25126421 (PMC11203698; doi:10.3390/ijms25126421)
Supplement: Supplementary file 1 [file ijms-25-06421-s001.zip › ijms-3023432-supplementary.pdf]

Electronic Supplementary Information

A Novel Boron Dipyrromethene-Erlotinib Conjugate for Precise Photodynamic Therapy against Liver Cancer

Wenqiang Wu [1], Cheng-Miao Luo [2], Chunhui Zhu [2], Zhengyan Cai [1,*] and Jian-Yong Liu [2,3,*]

[1] China State Institute of Pharmaceutical Industry, Pudong New Area,Shanghai 201203, China
[2] Key Laboratory of Molecule Synthesis and Function Discovery, Fujian Province University, College of Chemistry, Fuzhou University, Fuzhou 350108, China

3 State Key Laboratory of Photocatalysis on Energy and Environment & National & Local Joint Biomedical Engineering Research Center on Photodynamic Technologies, College of Chemistry, Fuzhou University, Fuzhou 350108, China

[*] Corresponding author. E-mail: caizy2007@163.com; lkw82@fzu.edu.cn.
Tel.: +86-591-22867105.

Table of Contents

Reagents and instruments

Photophysical and photochemical properties

Determination of lipid-water partition coefficients

Cell line and culture conditions

Animal feeding

Figure S1 Electronic absorption spectra of **6** and **7** in DMF

Figure S2 Fluorescence emission spectra of **6** and **7** in DMEM

Table S1 The pharmacokinetic properties of **6** and **7** predicted by the SwissADME webserver.

Figure S3 Cell viability of HepG2 under light irradiation at a dose of 1.5 $J\cdot cm^{-2}$.

Figure S4 H&E-stained images of main organs from the H22 tumor-bearing mice treated with saline and **6** + Laser

Figure S5 ^{1}H NMR spectrum of **4** in $CDCl_3$

Figure S6 ^{13}C NMR spectrum of **4** in $CDCl_3$

Figure S7 HRMS spectrum of **4**

Figure S8 ^{1}H NMR spectrum of **5** in $CDCl_3$

Figure S9 ^{13}C NMR spectrum of **5** in $CDCl_3$

Figure S10 HRMS spectrum of **5**

Figure S11 ^{1}H NMR spectrum of **6** in $CDCl_3$

Figure S12 HRMS spectrum of **6**

Figure S13 ^{1}H NMR spectrum of **7** in $CDCl_3$

Figure S14 ^{13}C NMR spectrum of **7** in $CDCl_3$

Figure S15 HRMS spectrum of **7**

Reagents and instruments

All the reactions were performed under an atmosphere of nitrogen and monitored by thin-layer chromatography (TLC). Toluene and dichloromethane (DCM) were distilled from sodium and calcium hydride, respectively. All the other agents and solvents were of analytical reagent (AR) and used as received. Column chromatographic purifications were carried out on silica gel (200-300 mesh, Anhui Liangchen Silicon Material Co., Ltd.). 3-(4,5-dimethylthiazol-2-yl)-2,5-Diphenyltetrazolium bromide (MTT) were obtained from Sigma-Aldrich. Dulbecco's Modified Eagle Medium (DMEM), fetal bovine serum (FBS), and antibiotics (penicillin and streptomycin) were purchased from Thermo Fisher Scientific. LysoTracker Green DND-26 and MitoTracker Green FM were purchased from Beyotime Institute of Biotechnology (Shanghai, China). Trypsin, penicillin-streptomycin liquid, and ROS assay kit (2',7'-dichlorofluorescein diacetate, DCFH-DA) were purchased from Ding Guo Prosperous (Beijing, China).

^{1}H NMR and ^{13}C NMR spectra were conducted on a AVANCE III spectrometer (^{1}H, 400 MHz; ^{13}C, 101 MHz) in $CDCl_3$. Chemical shifts (δ) were expressed in ppm relative to tetramethyl silane (TMS, δ = 0 ppm). High-resolution mass spectra (HRMS) analysis was carried out on an Agilent 6520 ACURATE-Mass Q-TOF Mass Spectra. Electronic absorption spectra were measured on a PerkinElmer Lambda 365 UV-visible absorption spectrometer. Fluorescence spectra were obtained on a Varian Cary Eclipse spectrometer. Intracellular fluorescence imaging was carried out on Olympus FV1000 confocal laser scanning microscope. The intracellular ROS levels were measured by microplate reader (SpectraMax® i3x). *In vivo* and *ex vivo* fluorescence images were obtained by FMT 2500LX Fluorescence Molecular Tomography system (PerkinElmer).

Photophysical and photochemical properties

Electronic absorption and fluorescence emission spectra

The samples (**6** or **7**) were dissolved in dimethylformamide (DMF) to give 1 mM stock solutions, which were then diluted in quartz colorimetric utensil with 3 mL DMF to obtain solutions with different concentrations (2, 4, 6, 8, 10, 12, 14, and 16 μM). Electronic absorption spectra were recorded at range of 300-800 nm. The molar extinction coefficient was calculated by Lambert-Beer law. Fluorescence emission spectra were collected from 620 to 800 nm under the excitation at 610 nm. The fluorescence quantum yields (Φ_F) in DMF were determined by the equation:

$$\Phi_F \text{ (sample)} = \Phi_F^{std} \frac{F_{sample} * A_{std} * \eta_{sample}^2}{F_{std} * A_{sample} * \eta_{std}^2}$$

where A, F, and η are the absorbance of sample at 610 nm, the measured fluorescence area under emission peak (λ_{ex} = 610 nm), and refractive index of the solvent, respectively. Here, the unsubstituted zin(II) phthalocyanine (ZnPc) in DMF was used as the reference (Φ_F^{std} = 0.28). To minimized the reabsorption of the radiation by ground-state species, the fluorescence spectra were obtained by adjusting the absorbances of samples at 610 nm to the range among 0.04-0.05.

Singlet oxygen ($^1O_2$) generation efficiency evaluation

The $^1O_2$ generation ability of PSs **6** and **7** was indirectly detected by using 1,3-diphenylisobenzofuran (DPBF) as the $^1O_2$ scavenger. Briefly, a mixture of PS and DPBF in DMF (with 0.04% CEL, pH 7.4) was illuminated with laser (λ = 660 nm, 20 mW/cm^2) for 10 minutes. Then the electronic absorption spectra of the mixtures were recorded at different time intervals at range of 300-800 nm. Degradation rates of DPBF was monitored by recording the absorption changes at 415 nm with illumination time, which suggested the $^1O_2$ generation ability.

Determination of lipid-water partition coefficients

10 mL of a saturated solution of compound **6** in n-octanol was added to a round-bottom flask, and then 10 mL of PBS buffer solution was poured carefully. The mixture was stirred gently for 24 hours. Subsequently, the aqueous and organic layers were separated and centrifuged to remove undissolved solids (12000 r/min, 10 min).

Aqueous phase testing procedure: Freeze-dry the aqueous phase, then dissolve the residue with 100 μL DMF. Measure the fluorescence intensity of the sample at 693 nm.

Organic phase testing procedure: 10 μL n-octanol solution was taken and mixed with 1 mL DMF. The fluorescence intensity of the sample at 693 nm was then detected.

The lipid-water partition coefficient of compounds **6** and **7** was calculated by the following formula.

$$Log\ P = Log\ \frac{F_o}{F_w}$$

F_o and F_w represents the fluorescence intensity of the organic phase and aqueous phase, respectively.

Cell line and culture conditions

The HepG2 cancer cells were purchased from the Shanghai Institutes of Biological Science, Chinese Academy of Science, which were maintained in DMEM supplemented with penicillin-streptomycin (1%, v/v) and fetal calf serum (10%, v/v) at 37 °C under a humidified 5% CO_2 atmosphere.

Animal Feeding

Female ICR mice (5-6 weeks) were purchased from the Laboratory Animals Services Center of Wushi (Fuzhou, Fujian, China) and raised under standard conditions. Animal experiments were approved by the Animals Experimentation Ethics Committee of Fuzhou University and carried out in accordance with the institutional guidelines.

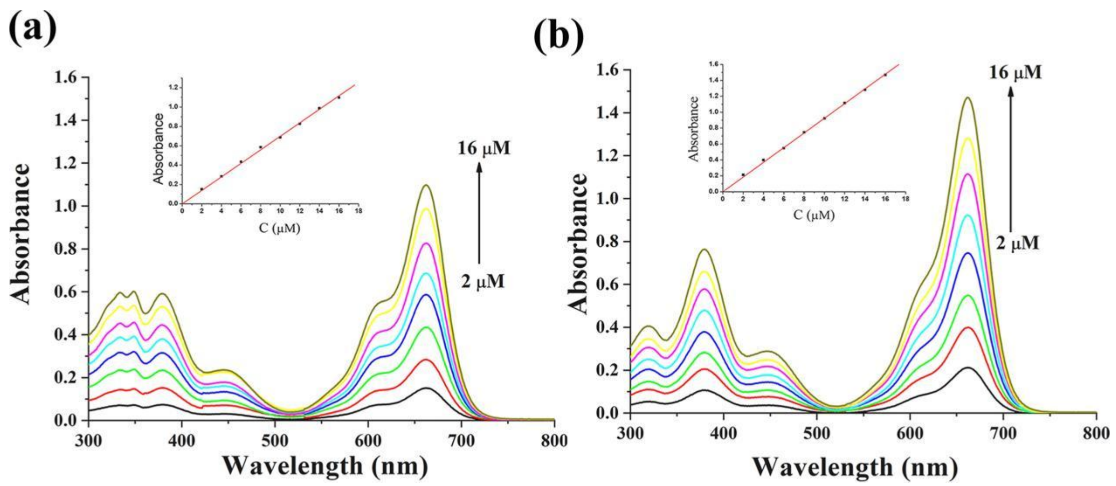

Figure S1. Electronic absorption spectra of **6** (a) and **7** (b) in DMF

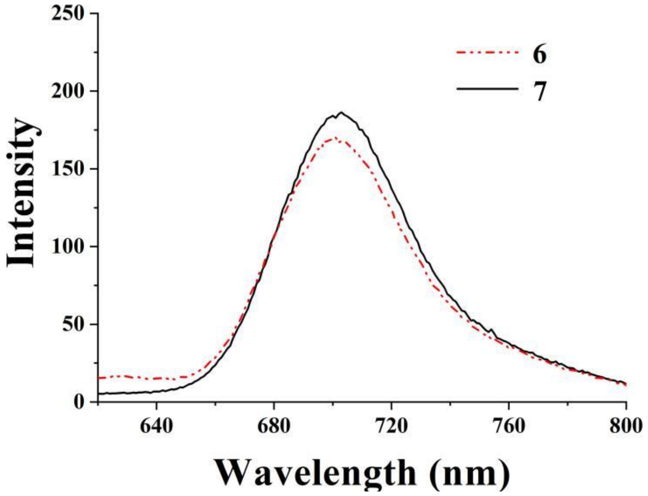

Figure S2. Fluorescence emission spectra of **6** and **7** in DMEM

Table S1. The pharmacokinetic properties of **6** and **7** predicted by the SwissADME webserver.

	**MW[a] (Da)**	**LogS[b]**	**LogP$_{o/w}$[c]**	**GI[d] absorption**	**BBB[e] permeant**
**6**	1624.12	-16.13	7.88	Low	No
**7**	1332.81	-14.29	7.24	Low	No

[a] Molecular Weight. [b] Solubility in aqueous solution. [c] Octanol-water partition coefficients. [d] Gastrointestinal adsorption. [e] Blood-brain-barrier permeant.

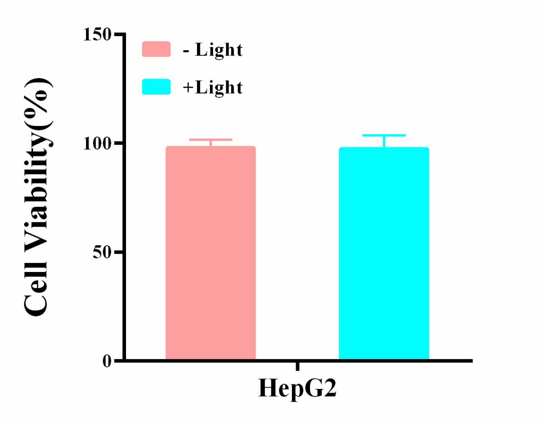

Figure S3. Cell viability of HepG2 under light irradiation at a dose of 1.5 J·cm^{-2}.

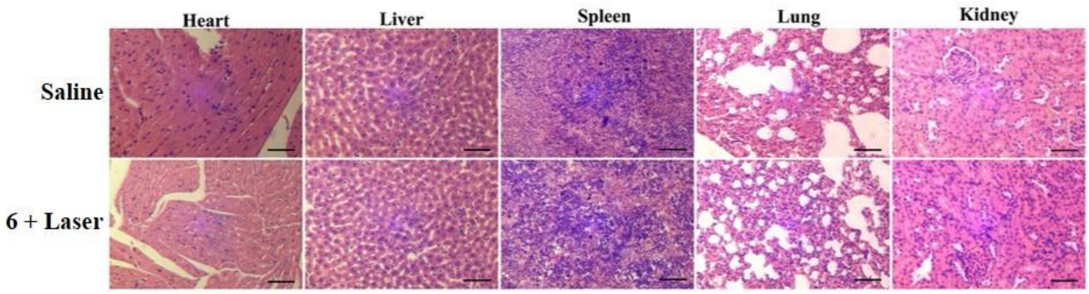

Figure S4. H&E-stained images of main organs from tumor-bearing mice treated with saline and **6** plus laser irradiation.

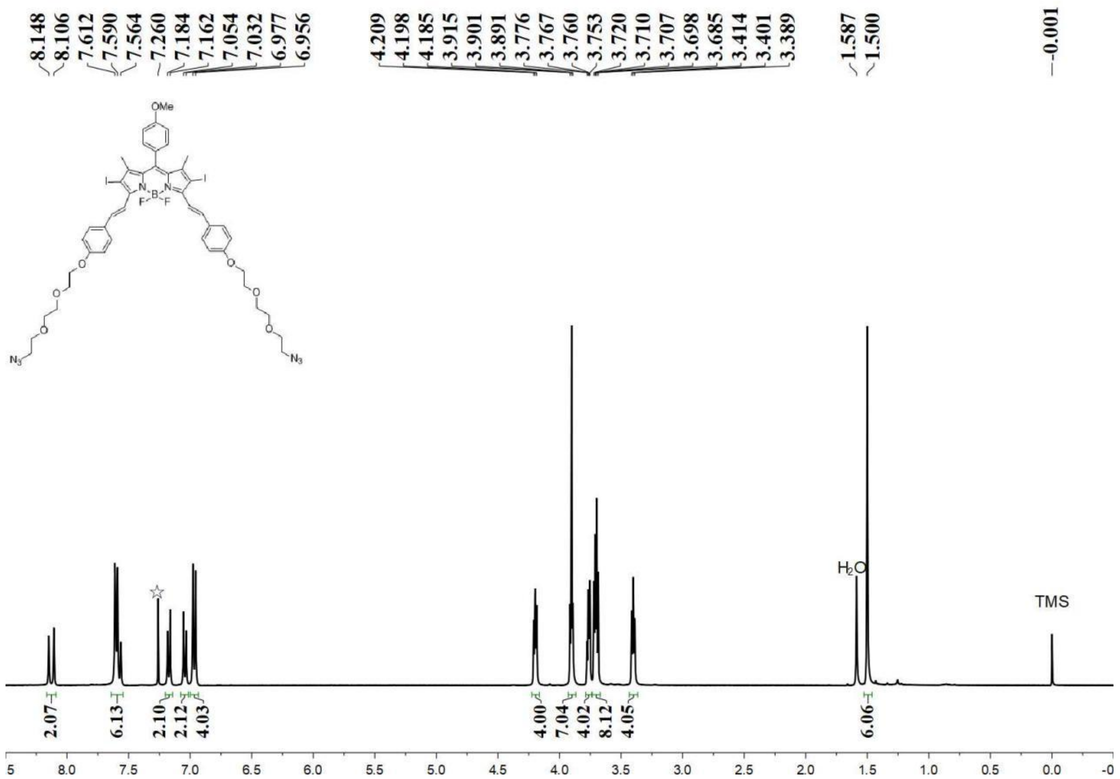

Figure S5. ^{1}H NMR spectrum of **4** in $CDCl_3$

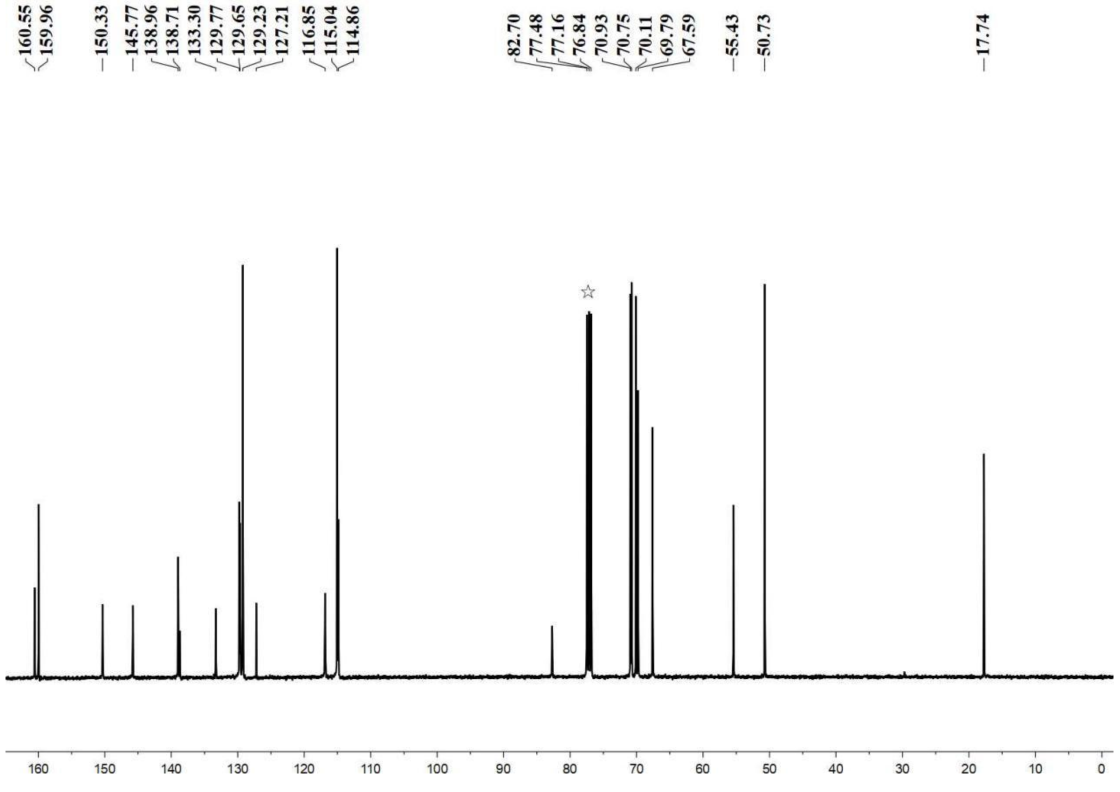

Figure S6. ^{13}C NMR spectrum of **4** in $CDCl_3$

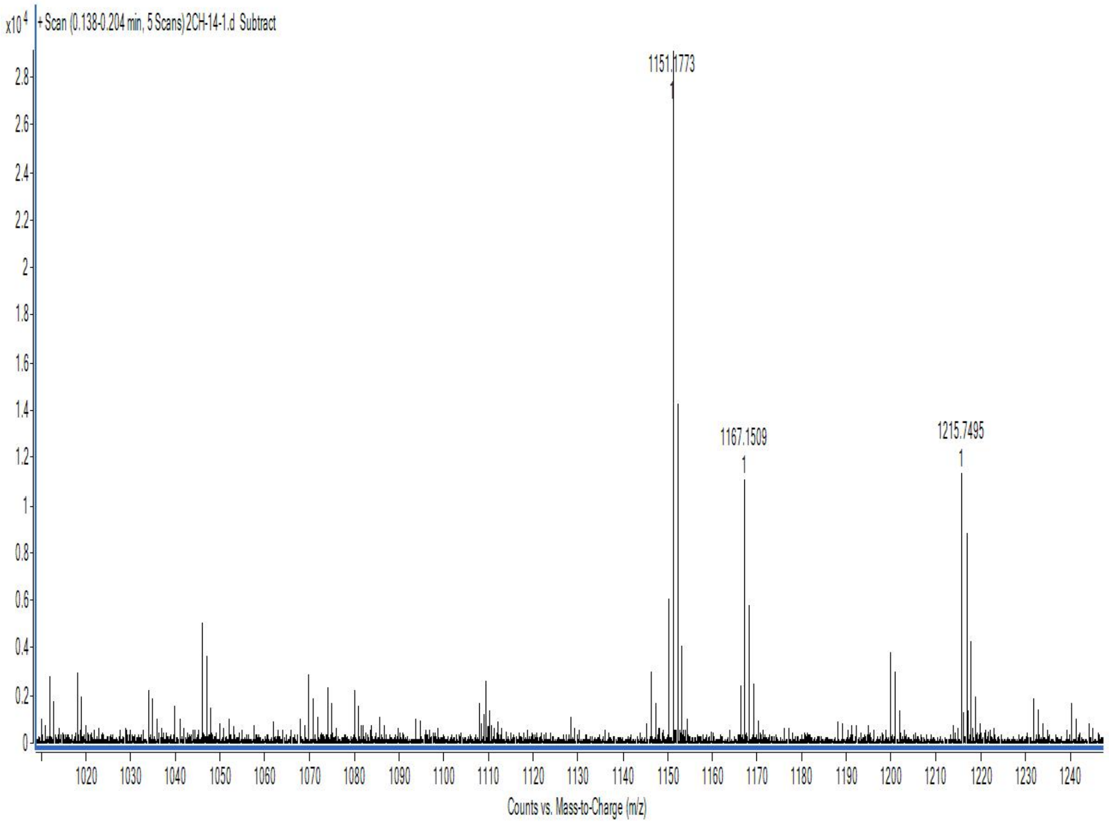

Figure S7. HRMS spectrum of **4**

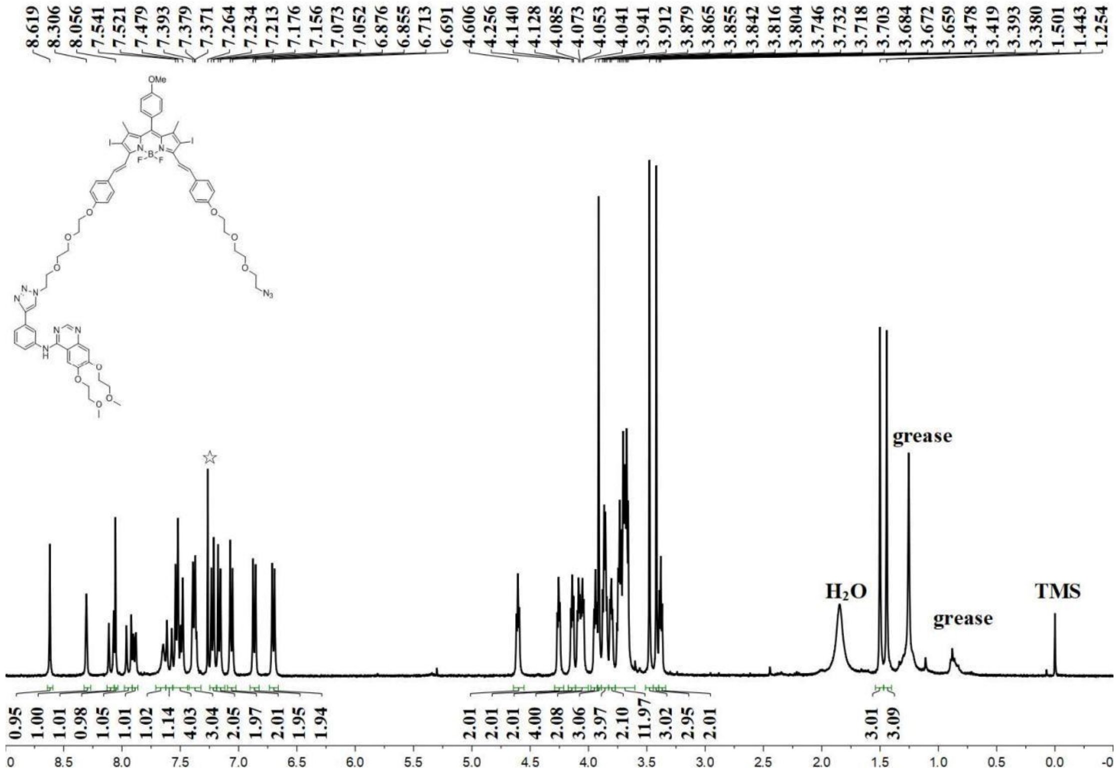

Figure S8. ^{1}H NMR spectrum of **5** in $CDCl_3$

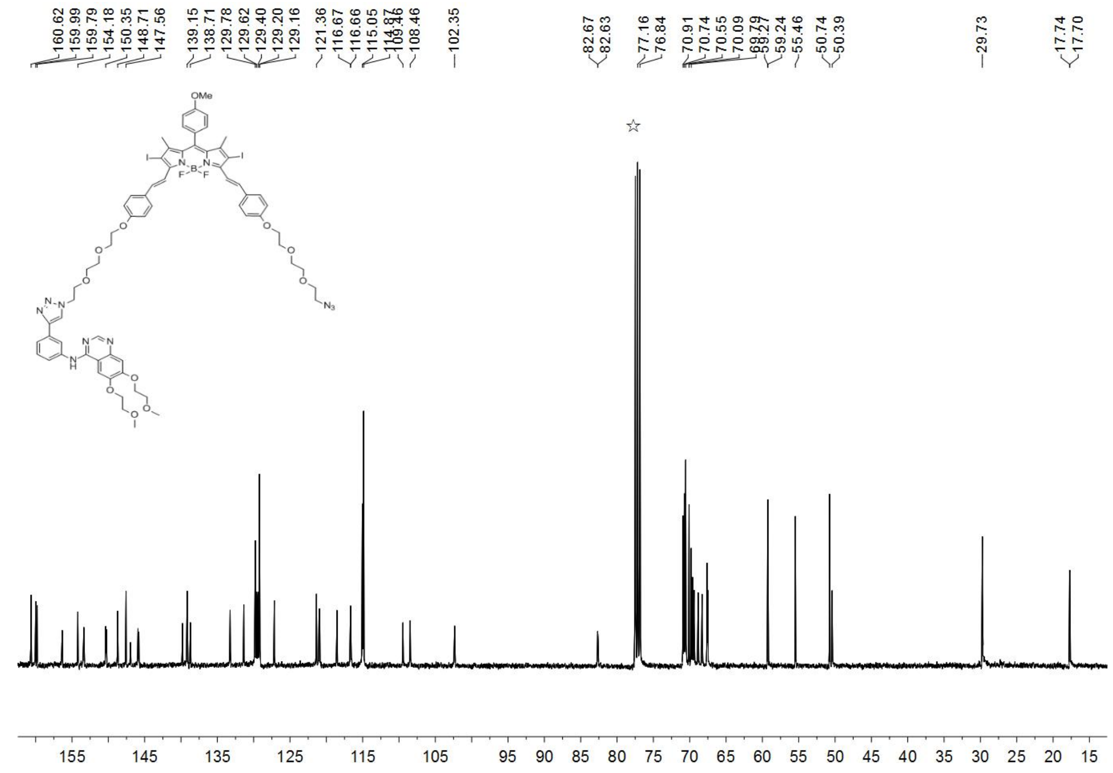

Figure S9. ^{13}C NMR spectrum of **5** in $CDCl_3$

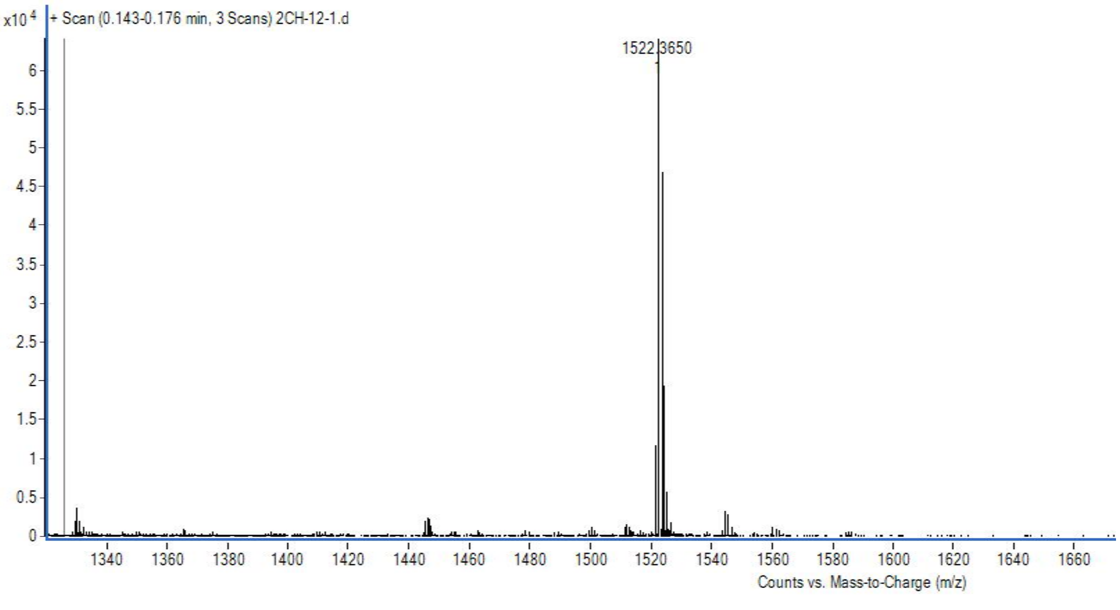

Figure S10. HRMS spectrum of **5**

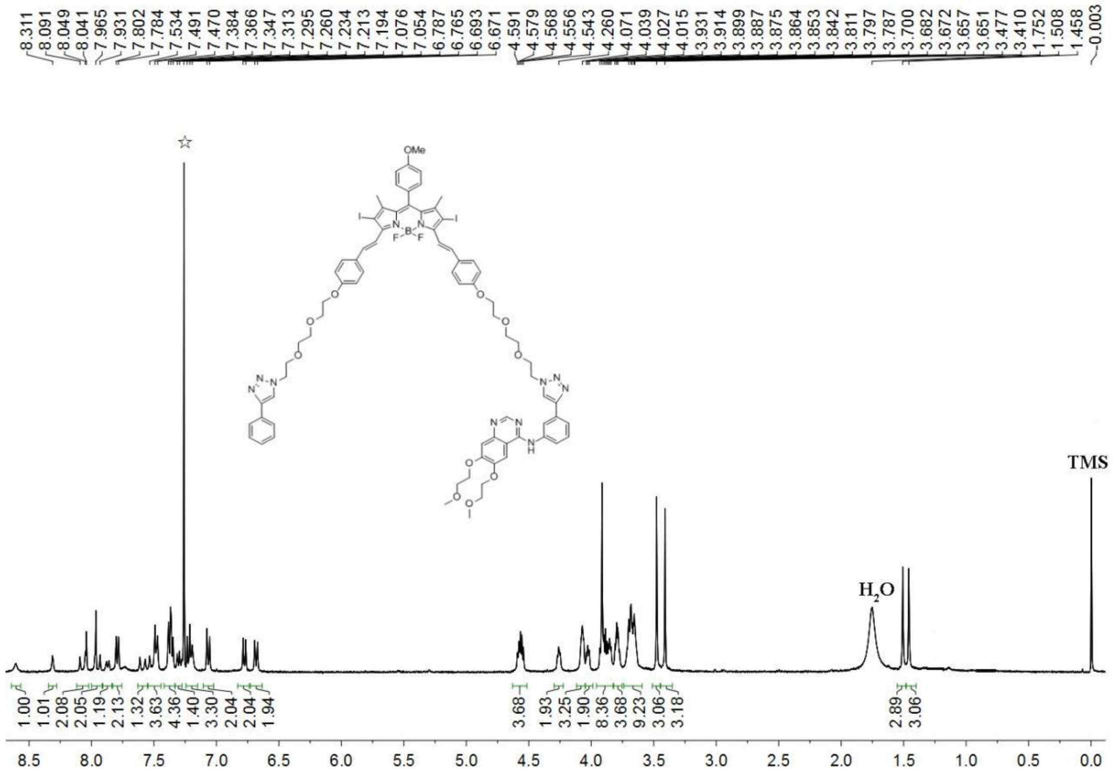

Figure S11. ^{1}H NMR spectrum of **6** in $CDCl_3$

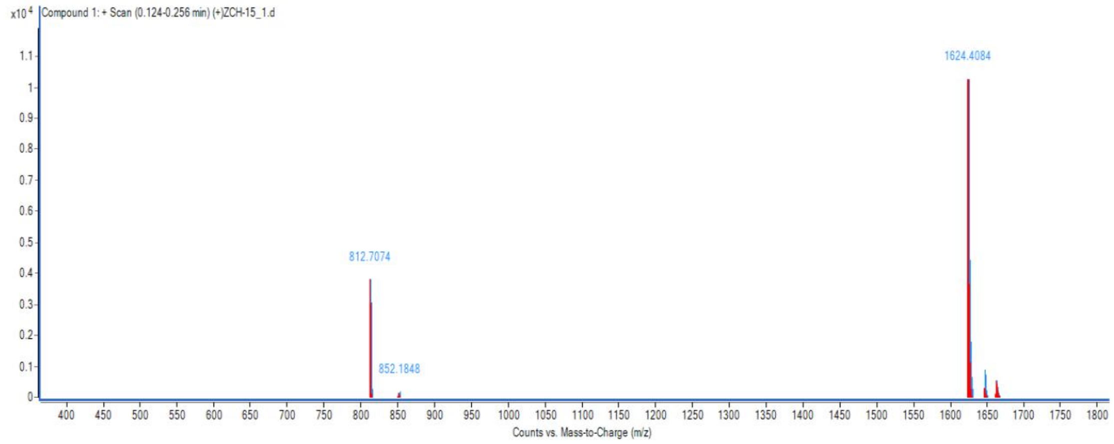

Figure S12. HRMS spectrum of **6**

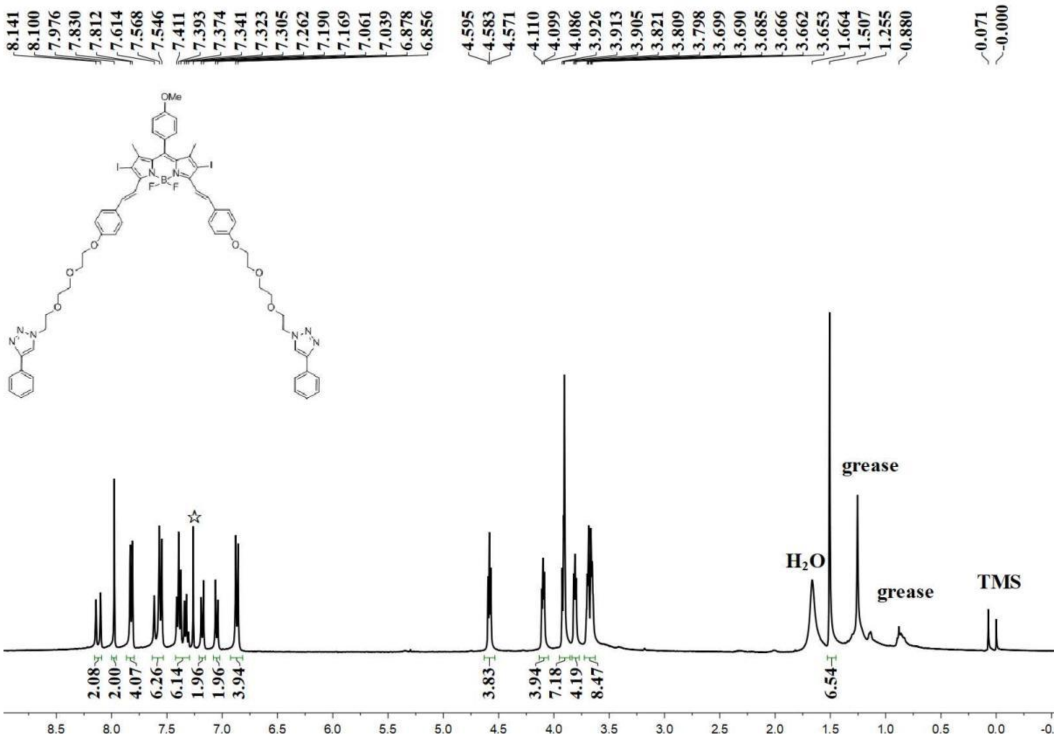

Figure S13. ^{1}H NMR spectrum of **7** in $CDCl_3$

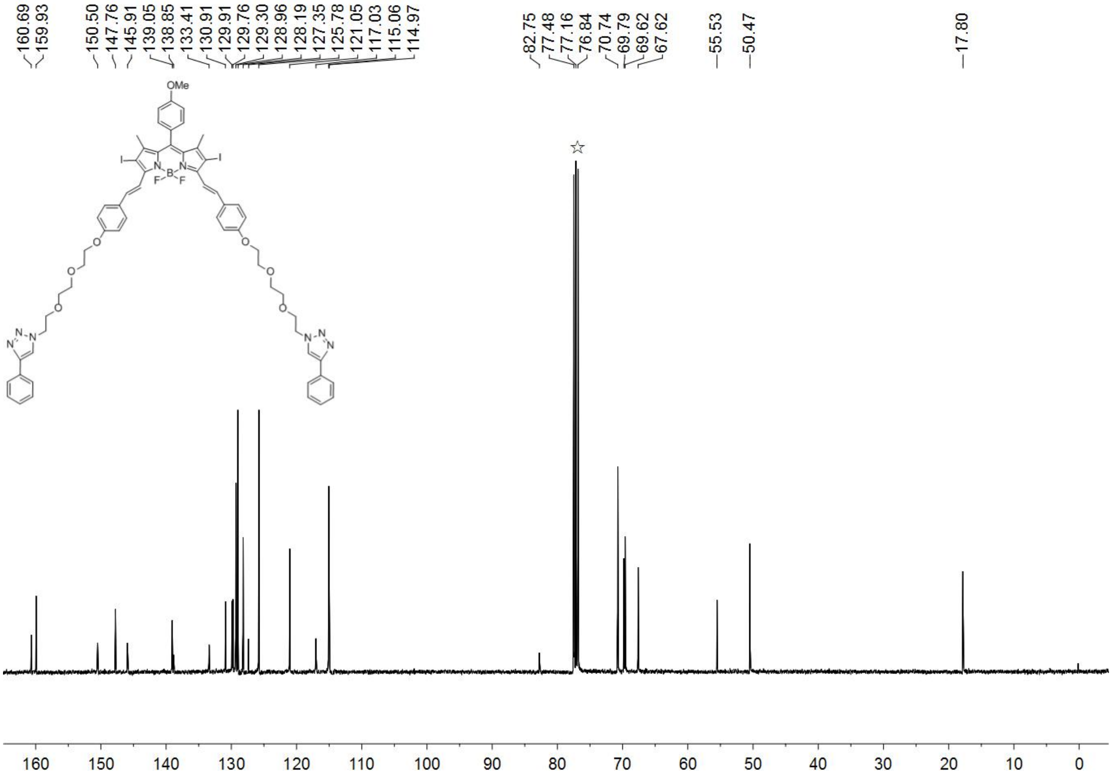

Figure S14. ^{13}C NMR spectrum of **7** in $CDCl_3$

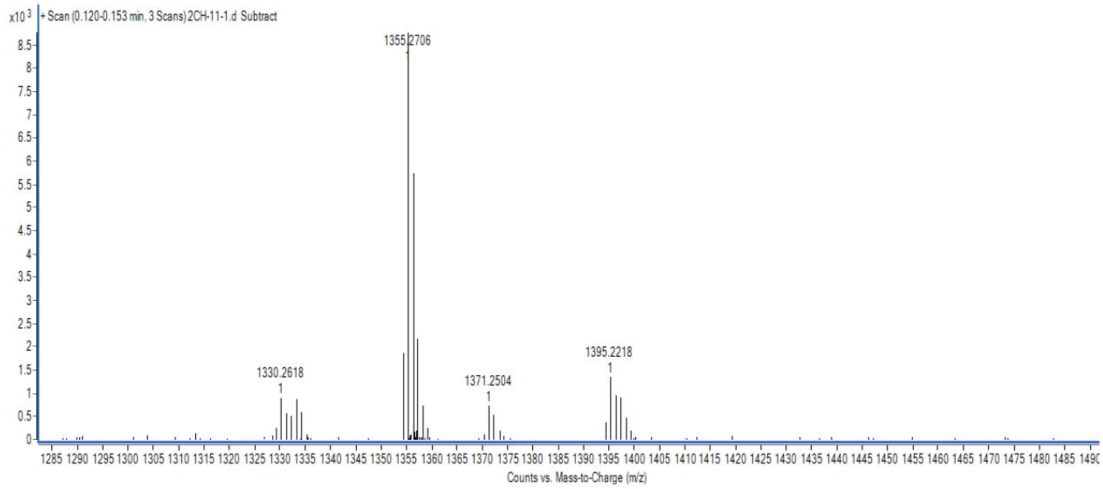

Figure S15. HRMS spectrum of **7**
